# Perfluorination of Aromatic Compounds Reinforce Their van der Waals Interactions with Rare Gases: The Rotational Spectrum of Pentafluoropyridine-Ne

**DOI:** 10.3390/molecules27010017

**Published:** 2021-12-21

**Authors:** Alberto Macario, Susana Blanco, Ibon Alkorta, Juan Carlos López

**Affiliations:** 1 Departamento de Química Física y Química Inorgánica, Facultad de Ciencias, IU CINQUIMA Universidad de Valladolid, 47011 Valladolid, Spain; alberto.macario@uva.es (A.M.); susana.blanco@uva.es (S.B.); 2 Instituto de Química Médica (CSIC), Juan de la Cierva 3, 28006 Madrid, Spain; ibon@iqm.csic.es

**Keywords:** van der Waals interactions, rotational spectroscopy, structure, computational chemistry, fluorine aromatic compounds

## Abstract

The rotational spectrum of the pentafluoropyridine-Ne complex, generated in a supersonic jet, has been investigated using chirped-pulse microwave Fourier transform spectroscopy in the 2–8 GHz range. The spectra of the ^20^Ne and ^22^Ne species have been observed, and the rotational constants have been used to determine the structure of the complex. This structure, and those of the previously experimentally studied complexes benzene-Ne and pyridine-Ne, are an excellent benchmark for the theoretical calculations on these adducts. These complexes and hexafluorobenzene-Ne have been investigated at the CCSD/6-311++G(2d,p) level. The calculations reproduce the experimental structures well and show how the van der Waals complexes are stronger for the perfluorinated compound.

## 1. Introduction

Supersonic jet expansions have been widely used to generate intermolecular complexes of diverse nature, which are studied by spectroscopic methods to characterize the noncovalent forces responsible for their formation. Fourier transform microwave spectroscopy (FTMW) combined with supersonic jets with reinforced high resolution and sensitivity [1,2,3,4] has contributed to the study of challenging molecular systems, including weakly bound molecular complexes [5]. This technique makes possible the unambiguous discrimination between different isomers, including tautomers, conformers, or isotopomers. The spatial mass distribution of such species has unique spectroscopic constants and distinct individual rotational spectra, a key feature that makes FTMW techniques powerful tools [6] in cases where other techniques may struggle. Complexes generated in supersonic expansions are isolated species that can be characterized in absence of intermolecular forces existing in condensed phases. This provides an ideal environment to investigate their intrinsic properties, structure, and interactions, which can be benchmarked with the results of theoretical calculations [7]. 

Aggregates formed by a molecule and a rare gas (RG) atom, generally that used to drive the expansion, have been studied to characterize the van der Waals interactions due to dispersion forces [8]. These forces have an important role in the stabilization of biological systems and materials [9], and have been also studied in complexes or rare gases present in the upper atmosphere with oxygen molecules in excited electronic states [10]. Aromatic rings offer a relatively large surface for dispersion interaction forces, so these kinds of molecules have often been used to model the interactions with rare gas atoms. Complexes of the prototype rings, benzene (BZ), with He [11], Ne [12,13], Ar [13,14], Kr [15], and Xe [13], or pyridine (PY), with He [16], Ne [17,18,19,20], Ar [19,21,22], and Kr [21], have been studied. Complexes of RG atoms with other six-membered ring aromatic molecules such as pyrimidine [23,24], pyridazine [25,26], and fluorobenzene [27,28,29] or fluoropyridine [30] derivatives have also been characterized.

The structure of molecule-RG atom complexes has been investigated through the observation of the rotational spectra of different isotopologues, including those of RG atoms. Although those parameters are often affected by intermolecular vibrations, it has been possible to investigate the geometry of the heterodimers, which in the case of the benzene-RG complex corresponds to a *C*_6v_ symmetry where the RG atom lies along the *C*_6_ benzene symmetry axis [13,14]. For pyridine-RG complexes, the RG atom is above the ring plane but is considerably shifted towards the N atom [16,17,18,19,20,21,22]. The rotation of the RG atom to explore both sides of the ring would give rise to two equivalent forms; however, with the exception of He [11,16], the barriers hindering this flipping motion are high enough to prevent the observation of tunneling effects. In many cases, under the assumption that the RG stretching motion can be isolated from bending vibrations, the dissociation energies of these complexes have been estimated from the *Δ*_J_ centrifugal distortion constants based on the pseudodiatomic approximation [17,20,22,23,24,25]. Additionally, in some cases, information about the bending motions has been obtained [24,31].

The complexes of perfluorinated aromatic rings such as hexafluorobenzene (F_6_BZ) or pentafluoropyridine (F_5_PY) with water [32,33] ammonia [34], and formaldehyde [35] have led to important conclusions about the changes induced by perfluorination in the aromatic π-cloud. The aromatic π-cloud acts, in general, as a Lewis base in noncovalent interactions. Fluorine atoms withdraw the electron density of the π-cloud region, creating a π-hole [36] which acts as a Lewis acid [37,38,39,40] that can interact with lone-pair-bearing atoms and enhance anion-π interactions [39,41,42]. In fact, in the complexes of F_6_BZ or F_5_PY with water, ammonia, or formaldehyde, the O or N lone pairs of these small molecules point directly toward the center of the ring, showing the existence of lone-pair···π-hole interactions. These interactions show almost equal contributions of electrostatic and dispersion forces [34,35], but the changes in the structures from O–H···π, N–H···π in the BZ–H_2_O or BZ–NH_3_ or N···C=O (n→ π*) in PY-CH_2_O to the H_2_O···π-hole, H_3_N···π-hole or H_2_C=O···π-hole in F_6_BZ and F_5_PY complexes can probably be attributed mainly to electrostatic changes. An investigation of the effects of perfluorination on the π-cloud and their implications on the dispersion forces contributing to the formation of complexes has not been conducted. In this paper we characterized the structure of the complex F_5_PY-Ne from the rotational spectrum. We compared the structure of this complex with previous experimental results on PY-Ne. Furthermore, a comparative study on the theoretically calculated energies and binding properties of BZ-Ne, PY-Ne, F_6_BZ-Ne and F_5_PY–Ne was carried out. 

## 2. Results and Discussion

### 2.1. Rotational Spectrum

**Figure 1:**
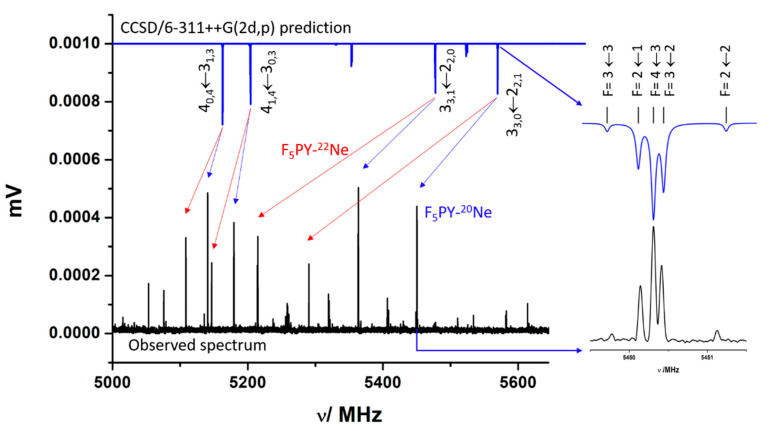
A section of the CP-FTMW spectrum in the 5.0–5.6 GHz region recorded from a supersonic jet expansion of F_5_PY diluted in Ne. The comparison with the prediction of the spectrum carried out from the rotational parameters predicted at CCSD/6-311++G(2d,p) (upper blue inverted trace) for F_5_PY-Ne complex helps to identify the patterns of the 4_0,4_←3_1,3_, 4_1,4_←3_0,3_ and 3_3,1_←2_2,0_, 3_3,0_←2_2,1_ doublets for both the parent (blue arrow) and ^22^Ne (red arrow) isotopologues. The excerpt compares the observed and predicted ^14^N quadrupole coupling hyperfine structure for the 3_3,0_←2_2,1_ transition where the components are identified by the quantum number F, taking values from (J + I) to (J − I) reflecting the coupling between the overall rotation moment (J) and ^14^N nuclear spin moments (I = 1).

We recently studied the broadband rotational spectrum of the complex F_5_PY-CH_2_O [35], generated in a supersonic jet driven by an expansion of Ne. In the spectrum, the most intense lines were those of the monomers, followed by those of the complex F_5_PY-H_2_O. Several sets of rotational lines having a similar intensity to those of F_5_PY-CH_2_O lines remained unassigned (see Figure S1 [35]). Those lines show the quadrupole coupling hyperfine structure (hfs) of a species carrying a ^14^N nucleus (see Figure 1). This hfs results from the interaction of the nuclear quadrupole moment of the ^14^N nucleus (*I* = 1), *eQ*, with the electric field gradient, *q*, at the site of the N nucleus. The corresponding spectroscopic constants, *χ*_αβ_ (*α*, *β* = *a*, *b*, *c*), are the elements of the nuclear quadrupole coupling tensor, set up in the inertial principal axis system representation, and are a direct measure of the electric field gradient at the nitrogen nucleus (*χ*_αβ_ = *eQq*_αβ_). FTMW spectroscopy has enough resolution to detect and resolve the individual quadrupole hyperfine components (see Figure 1). Once the lines of all known species were dropped from the spectrum, a search of possible species was conducted to identify the unknown spectra. Among the possible species, those corresponding to complexes of the molecules in the mixture with the carrier gas should be considered; in this case, the complex F_5_PY-Ne. Using the calculated rotational parameters for this complex, a prediction of the spectrum in the 2–8 GHz region was made. According to the predictions, this van der Waals complex has only the *μ*_b_ type spectrum. The results of the theoretical calculations were valuable in the identification of rotational lines based on the frequency patterns and quadrupole coupling hyperfine structure. The quadrupole coupling constants are related to the electronic environment of this nucleus, and in this case, strongly depend on the orientation of the nitrogen atom in the principal inertial axis system. Figure 1 shows the comparison of the predicted spectrum for F_5_PY-Ne (upper blue trace), with the observed one (black lower trace) in the 5.0–5.6 GHz range. In this figure, it is illustrated how the R-branch transitions J + 1_0,J + 1_ ← J_1,J_ and J + 1_1,J + 1_ ← J_0,J_ or J + 1_J + 1,1_ ← J_J,0_ and J + 1_J + 1,0_ ← J_J,1_ formed well-defined doublets which were identified from frequency and intensity patterns for the parent species, and for the ^22^Ne isotopologue with a natural abundance ratio ^22^Ne/^20^Ne = 1/3. In the excerpt, it can be seen how the comparison of the predicted and observed quadrupole coupling hyperfine structure patterns confirms the assignment.

The assignments of the complete *μ*_b_-type spectra were followed from successive fitting-prediction-measurement cycles. The analysis of the spectrum was conducted [43] using a Hamiltonian H = H_R_^(A)^ + H_Q_, where H_R_^(A)^ represents Watson’s A-reduced semirigid rotor Hamiltonian in the I^r^ representation [44] and H_Q_ is the nuclear quadrupole coupling interaction term [45]. The determinable spectroscopic parameters are the rotational constants, the centrifugal distortion constants, and the elements of the nuclear quadrupole coupling tensor ***χ***. Usually, for ^14^N only the diagonal elements of the tensor are determined. The experimental constants determined for the F_5_PY-^20^Ne and F_5_PY-^22^Ne isotopologues of the complex are collected in Table 1, where they are compared to those calculated at the CCSC/6-311++G(2d,p) level. The parameters calculated at other levels are listed in Table S1. The observed frequencies are collected in Tables S9 and S10.

### 2.2. Structure of the Complex

**Figure 2:**
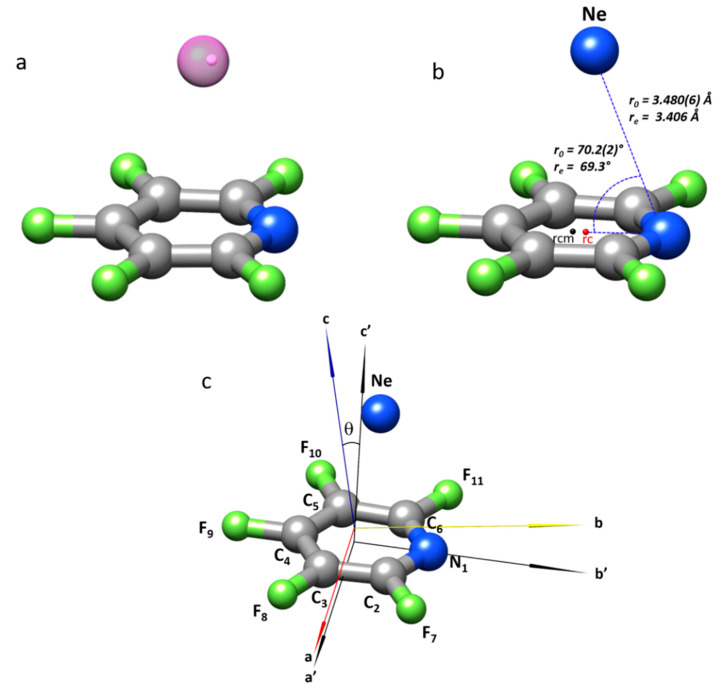
The structure of F_5_PY-Ne adduct: (**a**) The CCSD/6-311++G(2d,p) predicted structure is drawn with a translucent Ne atom to show the location of the Ne atom (white small sphere) according to the *r*_s_ coordinates. (**b**) The structure gives the values of the *r*_0_ and *r*_e_ (MP2/aug-cc-pVTZ) van der Waals bonding parameters. (**c**) The location of the F_5_PY and F_5_PY-Ne inertial axes is shown together with the definition of the angle θ. rcm is the F_5_PY center of mass and rc is the ring centroid.

The comparison of the rotational constants, the corresponding inertial *I*_α_ and planar moments *P*_αα_ (*α* = *a*, *b*, or *c*), and the quadrupole coupling constants *χ*_αα_ of F_5_PY-Ne with those of the monomer, F_5_PY, provides experimental insights into the structure of the complex (see Table 2 and Figure 2). F_5_PY is a planar molecule with *C*_2v_ symmetry. The *C*_2_ axis is coincident with the inertial axis *b* and the plane of the molecule is coincident with the *σ*_ab_ inertial plane, while the symmetry plane perpendicular to the molecule can be identified as the *σ*_bc_. In correspondence, the planar moment $P_{cc}=\left(I_{a}+I_{b}-I_{c}\right)/2=\underset{i}{\sum }m_{i}c_{i}^{2}$, giving the mass extension out of the *ab* inertial plane, is nearly zero for F_5_PY (see Table 2 and Figure 2, where the principal axes of F_5_PY are labeled with a prime). The changes in the rotational parameters upon complexation give interesting clues about the structure and symmetry of the F_5_PY-Ne adduct. *P*_cc_ is rather large for the cluster (see Table 2), indicating that Ne lies above (or below) the F_5_PY ring plane. The planar moment *P*_aa_, giving the mass extension out of the *bc* plane, is nearly the same for the monomer and the cluster (see Table 2). This proves that the Ne lies in the *σ*_bc_ molecular plane, which is preserved as a symmetry plane upon formation of the complex, thus giving a *C*_s_ symmetry of F_5_PY-Ne. Furthermore, the rotational constant *C* changes just about 1% indicating that the Ne atom lies close to the *c* inertial axis. It is worth noting that the *P*_aa_ values show a slight decrease when going from F_5_PY to F_5_PY-^20^Ne or F_5_PY-^22^Ne, revealing the contribution of large-amplitude intermolecular vibrations in the complex to this planar moment.

The *χ*_aa_, *χ*_bb_, and *χ*_cc_ quadrupole coupling constants shown in Table 2 are the diagonal elements of the quadrupole coupling tensor set up in the principal inertial axis system. For F_5_PY, due to the *C*_2v_ symmetry, the principal quadrupole coupling axis and inertial axis systems are coincident (i.e., *a*→*y*, *b*→*z*, *c*→*x*) [35,46], so the quadrupole coupling tensor is diagonal. The conservation of the *σ*_bc_ plane as a symmetry plane in the complex implies that the coincidence of the *a* inertial and *y* quadrupole coupling axes is also preserved in the complex. This can be deduced from the near equality of the *χ*_aa_ quadrupole coupling constant in F_5_PY and F_5_PY-Ne (see Table 2). The orientation of the *b* and *c* inertial axes of the aggregate results from the rotation of the monomer axes by an angle θ_bb’_ as defined in Figure 2. Using a value of θ_bb’_ = 11.0° to transform the experimental quadrupole coupling tensor constants of the monomer into those expected for the adduct, it gives *χ*_bb_ = −3.737 MHz and *χ*_cc_ = 1.771 MHz, values, very close to the experimental values of F_5_PY-^20^Ne (see Table 2). An angle of 13.1° gives *χ*_bb_ = −3.648 MHz and *χ*_cc_ = 1.682 MHz, close to the values of F_5_PY-^22^Ne. This evidences that the electric field gradient, and thus the electronic environment around the N atom, is not altered upon formation of the complex.

The degree of agreement between the experimental and calculated inertial data is better appreciated if we take the values of the planar moments of inertia $P_{\alpha \alpha }$. The three translational motions of the isolated Ne are replaced by three low-energy large-amplitude vibrational modes upon formation of the complex. Due to these van der Waals motions, which may add vibration-rotation interaction contributions to the moments of inertia, the usual methods for structure determination may supply poor results. One of these vibrations is a stretching *ν*_s_, and the other two, *ν*_ra_ and *ν*_rb_, can be associated to rotations of Ne around F_5_PY. For small displacements around the equilibrium structure these are described as nearly degenerate displacements of Ne in trajectories parallel to *a* (*ν*_ra_) and *b* (*ν*_rb_) axes. The frequencies calculated at B3LYP-D3BJ/6-311++G(2d,p) are *ν*_s_ = 53.1 cm^−1^, *ν*_ra_ = 22.2 cm^−1^, and *ν*_rb_ = 24.6 cm^−1^. The frequencies calculated with the MP2 method and the same basis set are *ν*_s_ = 48.1 cm^−1^, *ν*_ra_ = 18.4 cm^−1^ and *ν*_rb_ = 18.5 cm^−1^. It can be seen in Table 1, Table 2, and Table S1 that the planar moments *P*_aa_ and *P*_bb_ are well-described theoretically in correspondence with the good description of F_5_PY rotational parameters at the levels of calculation used (see Table S2) [35]. The main discrepancies come from the planar moment *P*_cc_, which is highly dependent on the position of the Ne atom relative to the ring. In this case, the best description is made at the CCSD level. Part of these discrepancies may arise from the intermolecular vibrational contributions, in particular stretching vibrations, which may result in larger *P*_cc_ experimental values corresponding with average ground vibrational state Ne-ring distances larger than those corresponding to equilibrium. As observed previously [35], the best agreement for the quadrupole coupling constants is obtained at MP2/6-311++G(2d,p) level.

The structure of F_5_PY-Ne has been further investigated from the analysis of the rotational parameters of the two observed isotopologues, by using the substitution (*r*_s_ [47,48]), effective (*r*_0_ [49]), and mass dependence (*r*_m_ [49,50]) methods, as illustrated in Figure 2. The substitution, *r*_s_, method compares the inertial moments of the parent and monosubstituted isotopologues through the Kraitchman equations [47] to give the absolute values of the substituted atom coordinates—in this case, Ne—in the principal inertial axis system of the parent molecule. The signs of the coordinates can be assigned from any reasonable geometry as effective (*r_0_)* or theoretical (*r*_e_) structures. This experimental approach has some limitations in locating atoms close to the principal inertial axes, or for light atoms such as hydrogen. The *r*_s_ coordinates of Ne in the complex are given in Table 3, where these are compared with those from the *r*_0_ method and theoretical CCSD calculations.

A total or partial effective *r_0_* structure can be obtained when bond distances and angles are obtained from a least-squares fit of the inertial moments of all observed isotopologues. For F_5_PY-Ne, we have assumed that the structure of F_5_PY does not change upon complexation, so the previously determined *r*_0_ structure [35] was kept fixed to determine the *r*(N-Ne) distance and ∠C_4_-N-Ne angle. The results are given in Table 3 and summarized in Figure 2, where it can be seen that the *r*_0_ value of the angle θ is in good agreement with that calculated from the quadrupole coupling parameters, if we take into account that these may be affected by the effects of intermolecular vibrations. The comparison of the experimental structures with those derived theoretically at the CCSD/6-311++G(2d,p) level shown in Table 3, or with other methods (see Table S3) reflect the same degree of agreement pointed out when comparing the experimental values of the planar moments (see above); the main discrepancies come from the distance between the Ne atom and the ring plane. The CCSD method gives the closest results and nicely reproduces the *r*_s_ values for F_5_PY-Ne. The CCSD predictions could then be considered reasonable, taking into account that the experimental *r*_0_ data might be more affected by intermolecular vibration contributions, giving rise to larger distances.

### 2.3. Comparison with Related Complexes

**Figure 3:**
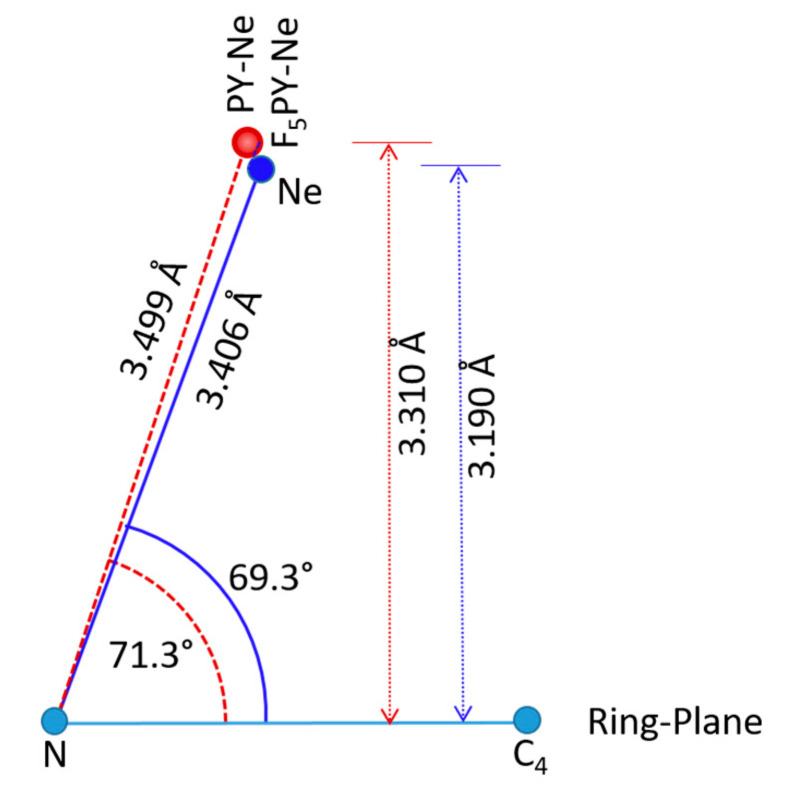
A sketch where the CCSD/6-311++G(2d,p) van der Waals predicted structure of F_5_PY-Ne (blue) is compared with that of PY-Ne (red) [20].

The experimental and CCSD geometry of F_5_PY-Ne is compared in Table 4 with those of the three related complexes, PY-Ne, F_6_BZ-Ne, and BZ-Ne. The geometrical parameters given are the distance R from Ne to the ring center of mass, and the angle φ between the line from Ne to the ring center of mass and a line perpendicular to the ring plane (Figure 3). These parameters allow a direct comparison with the geometries of two of these complexes, PY-Ne and BZ-Ne, already described experimentally. In all cases, the computational results reproduce well the *r*_s_ values for F_5_PY-Ne and the equilibrium *r*_e_ values derived from fitting the rotational parameters to Lennard-Jones type potential energy functions for the intramolecular stretching motions for PY-Ne [17] and BZ-Ne [13]. In the case of the *r*_0_ values, the experimental distances are always larger than the predicted equilibrium distances, and this fact may reflect the contribution of large-amplitude intermolecular vibrations, especially the stretching vibrations to the ground vibrational state parameters. The longest predicted intermolecular R distances are obtained for the complexes of the hydrogenated aromatic rings (PY-Ne and BZ-Ne). The perfluorination of the aromatic systems decreases the intermolecular distance around 0.1 Å. The changes in the structures of PY-Ne and F_5_PY-Ne are sketched in Figure 3. In both, the RG atom is shifted towards the N atom, but in F_5_PY-Ne it is closer to the ring plane. 

### 2.4. Binding Energies

The calculated binding energies at the CCSD(T) computational level and the DFT-SAPT contributions have been gathered in Table 5. As in the case of the intermolecular distances, the binding energies for the neon complexes of the hydrogenated aromatic systems are smaller than the perfluorinated ones by approx. 2 kJ mol^−1^. The DFT-SAPT partition shows that in all cases, dispersion is the most important contribution, with values between −3.1 and −3.9 kJ mol^−1^. The values of the dispersion term increase with the molecular weight of the aromatic system. A comparison of these values with those obtained with the empirical D3(BJ) method provides an excellent linear correlation (R^2^ = 0.99, see Figure S2). The second most important term is the repulsive exchange that also increases with the molecular weight of the aromatic system from 2.2 kJ mol^−1^ in PY-Ne to 3.3 kJ mol^−1^ in Bz-Ne. The other three attractive terms have small contributions with the following trend in absolute value in each complex: electrostatic > δHF > Induction.

**Figure 4:**
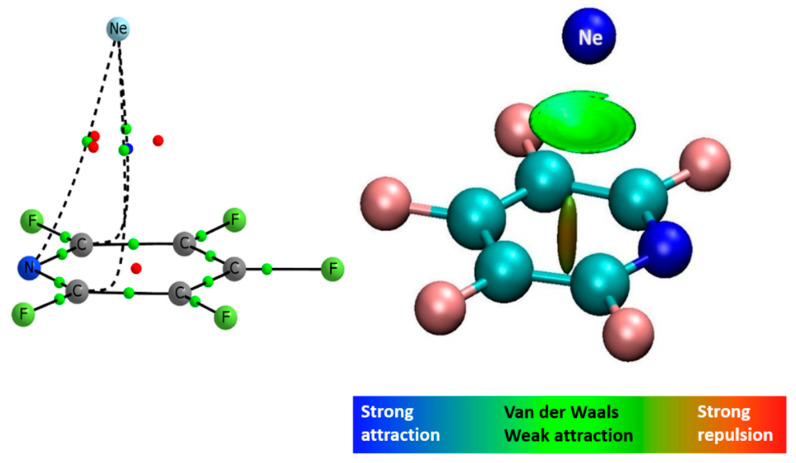
Molecular graph showing three bond critical points (**left**) and NCIPlot (**right**) of the F_5_PY-Ne complex.

The electron density analysis of the complexes shows the presence of three intermolecular BCPs in the F_5_PY-Ne complex (see Figure 4), one in the PY-Ne and six in the BZ-Ne and F_6_BZ-Ne ones (see the molecular graphs in Tables S5–S8). The values of the electron density of all the intermolecular BCPs are very close to what is considered to be the van der Waals limit in QTAIM theory (0.002 au). The largest value is obtained in the F_6_BZ-Ne complex (0.0027 au) and the smallest is obtained in the PY-Ne one (0.0022 au). Thus, the electron density values also are in concordance of very weak interactions between the two molecules. The NCIPlot analysis shows a region of weak interaction between the neon atom and the aromatic system (see Figure 4 for the F_5_PY-Ne complex).

## 3. Materials and Methods

### 3.1. Theoretical Methods

Starting values of the rotational parameters were obtained from optimization of the F_5_PY-Ne complex geometries using B3LYP-D3BJ/6-311++G(2d,p) [51,52,53], MP2/6-311++G(2d,p) [54,55], usually giving good predictions of the ^14^N quadrupole coupling constants [56], and MP2/aug-cc-pVTZ levels [57]. The results are collected in Table S1 of the supporting information. Finally, the geometry of the isolated molecules and complexes was optimized at the CCSD/6-311++G(2d,p) computational level. In order to improve the energetic description, a single point calculation at CCSD(T)/6-311++G(2d,p) [58] was performed using the previously obtained geometries. These calculations were carried out with the Gaussian-16 program [59]. The binding energy of the complexes was analyzed with the DFT-SAPT [60,61] method in the Molpro program [62], using the PBE0 functional [63] and the aug-cc-pVTZ basis set. The electronic characteristics of the complexes were analyzed with the quantum theory of atoms in molecules (QTAIM) [64] and the NCIPlot [65] using the CCSD/6-311++G(2d,p) wavefunction.

### 3.2. Experimental Methods

A commercial sample of F_5_PY was used. The spectrum of the F_5_PY-Ne complex was created in a supersonic jet of Ne and investigated using a chirped-pulse Fourier transform microwave spectrometer (CP-FTMW) [66], described elsewhere [67], and a narrowband Fabry–Perot Fourier transform microwave spectrometer (FP-FTMW) [68,69]. F_5_PY was kept at room temperature in a deposit inserted in the gas line close to the solenoid valve in both spectrometers. In the CP-FTMW instrument, which covers the 2–8 GHz frequency range, the spectra were recorded in steps of 2 GHz. The carrier gas was Ne at backing pressures of about 2 bar, expanding through a 0.8 mm nozzle in pulses of 700 μs duration. Chirp pulses of 4 μs were created by an arbitrary waveform generator and amplified to 20 W. The polarization signal was radiated from a horn antenna in a direction perpendicular to that of the expanding gas. A molecular transient emission spanning 40 μs was then detected through a second horn, recorded with a digital oscilloscope, and Fourier-transformed to the frequency domain. The accuracy of frequency measurements was higher than 10 kHz. In the FP-FTMW instrument, operated in the 5–13 GHz frequency range, Ne was also used at stagnation pressures ranging up to 2 bar, expanding in pulses of about 800 μs through a 0.8 mm nozzle. Short (typ. 0.3 μs, 10–300 mW) microwave pulses were used for polarization purposes. Typically, a ca. 400 μs-length time-domain spectrum was recorded at 40–100 ns intervals and converted to the frequency domain by a fast Fourier transformation. Due to the collinear arrangement of the jet and resonator axis, each rotational transition split into two Doppler components, so the resonant frequencies were taken as the arithmetic mean of both components. Frequency accuracy was lower than 3 kHz. The rotational spectra of the ^22^Ne isotopologue was observed in its natural abundance.

## 4. Conclusions

In this work, we studied the rotational spectrum of the complex F_5_PY-Ne to characterize its structure and properties. These data, together with those from the rotational spectra of the complexes of BZ [12,13] and PY [17,18,19,20] with Ne, were used as a benchmark for the theoretical data obtained on these complexes using CCSD/6-311++G(2d,p) level with excellent results. The calculations were extended to the complex F_6_BZ-Ne to investigate the effects of perfluorination of BZ and PY in the interaction of these compounds with Ne. The experimental and theoretical results show that the van der Waals distances are shorter by ca. 0.1 Å in the perfluorinated compounds, indicating the latter are strongly bound. The binding energies calculated for the complexes corroborate this conclusion, since the energies of BZ-Ne and PY-Ne (−2.9 kcal mol^−1^) are smaller than the values (−4.9 kcal mol^−1^) of perfluorinated compounds.

## Figures and Tables

**Table 1:** Experimental rotational parameters for the F_5_PY-^20^Ne and F_5_PY-^22^Ne isotopologues and their comparison to the predicted CCSD/6-311++G(2d,p) values for the parent species (Figure 2).

	F5PY-^20^Ne		F5PY-^22^Ne
**Param. ^a^**	exp	CCSD	exp
*A*/MHz	940.26871(21) ^b^	962.2	911.57618(20)
*B*/MHz	769.84484(19)	780.8	752.84325(17)
*C*/MHz	617.16139(13)	618.2	615.66886(11)
*κ*	−0.05	−0.05	−0.07
*P*_aa_/uÅ^2^	468.93082(23)	469.7	468.87713(21)
*P*_bb_/uÅ^2^	349.94576(23)	347.7	351.98461(21)
*P*_cc_/uÅ^2^	187.53784(23)	177.5	202.41668(21)
*Δ*_J_/kHz	0.4055(25)		0.4890(23)
*Δ*_JK_/kHz	6.329(10)		6.1360(91)
*Δ*_K_/kHz	−5.5700(68)		−5.4288(59)
*δ*_J_/kHz	0.1045(12)		0.1066(11)
*δ*_K_/kHz	−4.2430(84)		−3.9140(73)
3/2(*χ*_aa_)/MHz	2.9536(29)	3.11	2.9595(26)
1/4(*χ*_bb_-*χ*_cc_)/MHz	−1.37697(75)	−1.51	−1.33170(67)
			
*χ*_aa_/MHz	1.9687(19)	2.07	1.9730(17)
*χ*_bb_/MHz	−3.7383(25)	−4.05	−3.6499(22)
*χ*_cc_/MHz	1.7696(25)	1.98	1.6769(22)
			
*n*	139		118
*σ*/kHz	3.2		2.6

^a^ *A*, *B*, and *C* are the rotational constants. *κ* is the Ray asymmetry parameter *κ* = (2*B − A − C*)/(*A − C*). *P*_αα_ (*α* = a, b, c) are the planar moments of inertia, derived from the inertial moments *P*_cc_ = (*I*_a_ + *I*_b_ − *I*_c_)/2. *Δ*_J_, *Δ*_JK_, *Δ*_K_, *δ*_J_, and *δ*_K_, are the quartic centrifugal distortion constants. *χ*_aa_, *χ*_bb,_ and *χ*_cc_ are the ^14^N quadrupole coupling constants. *n* is the number of quadrupole coupling components fitted. *σ* is the rms deviation of the fit. ^b^ Standard errors in parentheses in units of the last digit.

**Table 2:** Comparison of the rotational constants (*A*, *B*, *C*), the moments of inertia (*I*_a_, *I*_b_, *I*_c_), and the planar moments (*P*_aa_, *P*_bb_, *P*_cc_) of F_5_PY and F_5_PY-Ne.

Parameters	F_5_PY ^a^	F_5_PY-^20^Ne	F_5_PY-^22^Ne
*A*/MHz	1481.58184(19) ^b^	940.26871(21)	911.57618(20)
*B*/MHz	1075.37335(17)	769.84484(19)	752.84325(17)
*C*/MHz	623.11194(16)	617.16139(13)	615.66886(11)
			
*I*_a_/uÅ^2^	341.107724(44)	537.80275(12)	554.73049(12)
*I*_b_/uÅ^2^	469.956791(74)	656.85847(16)	671.69241(15)
*I*_c_/uÅ^2^	811.05653(21)	819.36283(17)	821.34916(15)
			
*P*_aa_/uÅ^2^	469.95280(16)	468.93082(23)	468.87713(21)
*P*_bb_/uÅ^2^	341.10373(16)	349.94576(23)	351.98461(21)
*P*_cc_/uÅ^2^	0.00399(16)	187.53784(23)	202.41668(21)
			
*χ*_aa_/MHz	1.9664(53)	1.9687(19)	1.9730(17)
*χ*_bb_/MHz	−3.9534(72)	−3.7383(25)	−3.6499(22)
*χ*_cc_/MHz	1.9870(72)	1.7696(25)	1.6769(22)

^a^ Taken from reference [35]. ^b^ Standard errors in parenthesis in units of the last digit.

**Table 3:** Principal inertial axis coordinates for the neon atom of pentafluoropyridine···Ne complex. The table compares the substitution (*r*_s_) and effective (*r*_0_) coordinates with those (*r*_e_) calculated at the CCSD/6-311++G(2d,p) (CCSD) computational methods. The table also compares the effective bonding parameters determined from a least-squares fit of the observed rotational constants, and the angle θ of rotation between the principal inertial axis systems of F_5_PY and F_5_PY-Ne.

r_s_ Coordinates	*a*	*b*	*c*
$\left|r_{s}\right|$	[0.0000] ^a^	0.9672(15) ^b^	2.75959(54)
$r_{0}$	0.0000	0.911(23)	2.8134(64)
$r_{e}^{CCSD\;}$	0.0000	0.814	2.757022
**Parameters**	*r*_0_	$r_{e}^{\mathrm{CCSD}}$	*r*_s_
***r*** **(N···Ne)/Å**	3.480(6)	3.359	
**∠** **(CNNe)/°**	70.2(2)	69.2	
***r*(rc···Ne) ^c^/Å**	3.278(8)	3.192	
**R = *r*(rcm···Ne) ^d^/Å**	3.307(9)	3.215	3.260
**∠** **(Ne···rc···N)/°**	86.8(5)	86.8	
**∠** **(Ne···rcm···N)/°**	82.8(5)	82.2	
**φ ^e^/°**	7.2(5)	7.2	8.1
**θ** **^f^/°**	9.8	8.6	
**Fit**	experimental	Residuals ^g^	
**^20^** **Ne A/MHz**	940.26871(21)	−1.56	
**^20^** **Ne B/MHz**	769.84484(19)	0.41	
**^20^** **Ne C/MHz**	617.16139(13)	0.60	
**^22^** **Ne A/MHz**	911.57618(20)	−0.84	
**^22^** **Ne B/MHz**	752.84325(17)	1.13	
**^22^** **Ne C/MHz**	615.66886(11)	0.47	

^a^ The coordinate in square brackets is fixed to zero due to symmetry. ^b^ Estimated errors are given in parentheses in units of the last digit calculated according to ref [48]. ^c^ Distance from Ne to the ring-centroid (rc, see Figure 3). ^d^ Distance from Ne to the F_5_PY center of mass (rcm, see Figure 3).^e^ angle between the line from Ne to the ring center of mass (rcm) and a line perpendicular to the ring. ^f^ see Figure 2 for definition. ^g^ Differences between the experimental constants and those calculated from the determined *r*_0_ structure.

**Table 4:** Intermolecular distance between the center of mass of the aromatic system and the Neon atom (Å), R, and the deviation of the perpendicular axes (°), φ. The values from experimental data sources are provided in parenthesis.

Param.	Method	F_5_PY-Ne	PY-Ne	F_6_BZ-Ne	BZ-Ne
R/Å	CCSD	3.215	3.325	3.199	3.313
	(*r*_s/e_*, r*_0_)	(3.260 ^a^, 3.302)	(3.316 ^b^, 3.400 ^c^)		(3.2989 ^d^, 3.462 ^e^)
φ/°	CCSD	7.8	4.6	0.0	0.0
	(*r*_s/e_ *, r*_0_)	(7.2 ^a^, 8.1)	(4.2 ^b^, 6.2 ^c^)		(0.0 ^d^, 0.0 ^e^)

^a^*r*_s_ value, this work. ^b^ Equilibrium values corresponding to a study of van der Waals motions [17]. ^c^ Ref. [20]. ^d^ Equilibrium values corresponding to a study of van der Waals motions Ref. [13]. ^e^ Ref. [13].

**Table 5:** Bonding energies (BE in kJmol^−1^) calculated at the CCSD(T) level and DFT-SAPT contributions for the complexes PY-Ne, BZ-Ne, F_5_PY-Ne, and F_6_BZ-Ne.

	BE[CCSD(T)]	Elect.	Exchange	Induction	Dispersion	δHF
PY-Ne	−2.9	−0.66	2.21	0.02	−3.05	−0.10
BZ-Ne	−2.8	−0.81	2.67	0.01	−3.27	−0.14
F_5_PY-Ne	−4.9	−0.87	2.91	−0.04	−3.74	−0.13
F_6_BZ-Ne	−4.9	−1.03	3.33	0.01	−3.87	−0.16

## Data Availability

Data supporting reported results can be found as supplementary material available online. Any other data concerning this research can be requested from the authors.

## Supplementary Materials

The following are available online, Complete reference [59]; Figure S1: Observed broadband FTMW spectrum (2–8 GHz) of a mixture of C_5_F_5_N (*) and CH_2_CO (°) (reference [35]). Figure S2: Correlation between the dispersion component of the DFT-SAPT energy and the D3(BJ) correction; Table S1: Predicted Rotational parameters for the perfluoropyridine···Ne complex at different levels of theory; Table S2: Observed and predicted perfluoropyridine rotational parameters [34]; Table S3: Principal inertial axis *r*_s_ coordinates of the neon atom of pentafluoropyridine···Ne complex; Table S4: *r*_0_ geometry of pentafluoropyridine···Ne complex; Tables S5–S8: Molecular graphs, Optimized geometries, and energies at CCSD/6-311++G(2d,p) computational level for the complexes studied in this work; Tables S9 and S10: Observed rotational transitions and residuals of the parent and ^22^Ne isotopologues.

## Sample Availability

Samples of the compounds are commercially available.
