# Supplementary material for: Perfluorination of Aromatic Compounds Reinforce Their van der Waals Interactions with Rare Gases: The Rotational Spectrum of Pentafluoropyridine-Ne
Source: Molecules. 2021 Dec 21;27(1):17. doi: 10.3390/molecules27010017 (PMC8746256; doi:10.3390/molecules27010017)
Supplement: Supplementary file 1 [file molecules-27-00017-s001.zip › Supplementary materials-1477360.pdf]

Perfluorination of aromatic compounds reinforce their van der Waals interactions with rare gases: The rotational spectrum of pentafluoropyridine-Ne

Alberto Macario [1], Susana Blanco [1], Ibon Alkorta [2] and Juan Carlos López [1,*]

[1] Departamento de Química Física y Química Inorgánica, Facultad de Ciencias, IU CINQUIMA
Universidad de Valladolid, 47011 Valladolid (Spain), E-mail: alberto.macario@uva.es, susana.blanco@uva.es, juancarlos.lopeza@uva.es
[2] Instituto de Química Médica (CSIC), Juan de la Cierva 3, 28006 Madrid (Spain), ibon@iqm.csic.es
* Correspondence: juancarlos.lopeza@uva.es; Tel.: +34-983185891

Complete Reference [59]

Figure S1. Observed broadband FTMW spectrum (2-8 GHz) of a mixture of C_5F_5N (*) and CH_2CO (°) (reference [35]).

Figure S2. Correlation between the dispersion component of the DFT-SAPT energy and the D3(BJ) correction (kJ mol^{-1}).

Table S1. Rotational parameters predicted at B3LYP-D3BJ/6-311++G(2d,p) (DFT), MP2/6-311++G(2d,p) (MP2 1) MP2/aug-cc-pVTZ (MP2 2), and CCSD/6-311++G(2d,p) (CCSD) levels of theory for the perfluoropyridine···Ne complex.

Table S2. Observed rotational parameters of pentafluoropyridine and those predicted at B3LYP-D3BJ/6-311++G(2d,p) (DFT), MP2/6-311++G(2d,p) (MP2 1) MP2/aug-cc-pVTZ (MP2 2), and CCSD/6-311++G(2d,p) (CCSD) levels of theory (data taken from reference [35].

Table S3. Principal inertial axis coordinates for the neon atom of pentafluoropyridine···Ne complex, analyzing the spectra with a Watson´s semirigid Hamiltonian with the A reduction and the I^r representation. The table compares the equilibrium coordinates (r_e) calculated at the B3LYP-D3BJ/6-311++G(2d,p) (DFT), MP2/6-311++G(2d,p) (MP2 1), MP2/aug-cc-pVTZ (MP2 2) and CCSD/6-311++G(2d,p) (CCSD) levels of theory with the experimental substitution (r_s) and effective (r_0) structures.

Table S4. r_0 reometry (Å, atandard errosr in parentheses) for pentafluoropyridine-Ne (see figure 2).

Table S5. Molecular graph, Optimized geometry (Å) and energy (hartree) at CCSD/6-311++G(2d,p) computational level for pentafluoropyridine-Ne

Table S6. Molecular graph, Optimized geometry (Å) and energy (hartree) at CCSD/6-311++G(2d,p) computational level for pyridine-Ne

Table S7. Molecular graph, Optimized geometry (Å) and energy (hartree) at CCSD/6-311++G(2d,p) computational level for hexafluorobenzene-Ne

Table S8. Molecular graph, Optimized geometry (Å) and energy (hartree) at CCSD/6-311++G(2d,p) computational level for benzene-Ne

Table S9. Observed rotational transitions and residuals (all the values in MHz) for the pentafluoropyridine···Ne^{20} complex in the ground vibrational state.

Table S10. Observed rotational transitions and residuals (all the values in MHz) for the pentafluoropyridine···Ne^{22} complex in the ground vibrational state.

Complete Reference [59]

Gaussian 16, Revision A.03, Frisch, M. J.; Trucks, M. J.; Schlegel, H. B.; Scuseria, G. E.; Robb, M. A.; Cheeseman, J. R.; Scalmani, G.; Barone, V.; Petersson, G. A.; Nakatsuji, H.; Li, X.; Caricato, M.; Marenich, A. V.; Bloino, J.; Janesko, B. G.; Gomperts, R.; Mennucci, B.; Hratchian, H. P.; Ortiz, J. V.; Izmaylov, A. F.; Sonnenberg, J. L.; Williams-Young, D.; Ding, F.; Lipparini, F.; Egidi, F.; Goings, J.; Peng, B.; Petrone, A.; Henderson, T.; Ranasinghe, D.; Zakrzewski, V. G.; Gao, J.; Rega, N.; Zheng, G.; Liang, W.; Hada, M.; Ehara, M.; Toyota, K.; Fukuda, R.; Hasegawa, J.; Ishida, M.; Nakajima, T.; Honda, Y.; Kitao, O.; Nakai, H.; Vreven, T.; Throssell, K.; Montgomery Jr., J. A.; Peralta, J. E.; Ogliaro, F.; Bearpark, M. J.; Heyd, J. J.; Brothers, E. N.; Kudin, K. N.; Staroverov, V. N.; Keith, T. A.; Kobayashi, R.; Normand, J.; Raghavachari, K.; Rendell, A. P.; Burant, J. C.; Iyengar, S. S.; Tomasi, J.; Cossi, M.; Millam, J. M.; Klene, M.; Adamo, C.; Cammi, R.; Ochterski, J. W.; Martin, R. L.; Morokuma, K.; Farkas, O.; Foresman, J. B.; Fox, D. J. Gaussian, Inc., Wallingford CT, 2016.

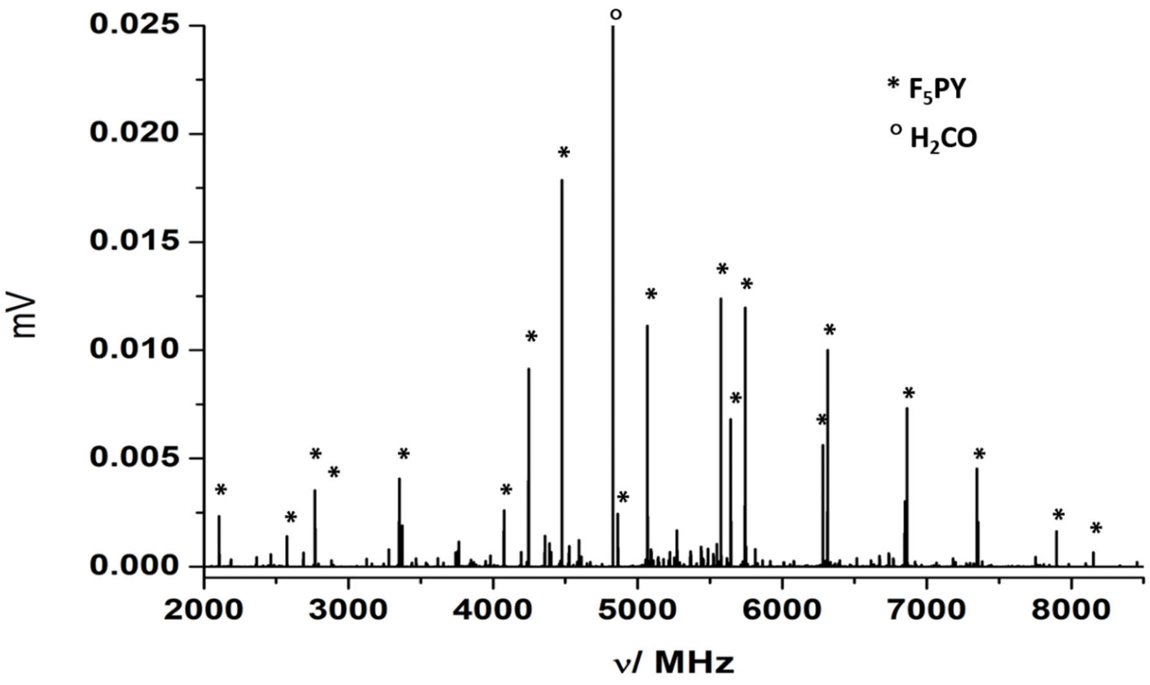

Figure S1. Observed broadband FTMW spectrum (2-8 GHz) of a mixture of F_5PY (*) and CH_2CO (º) (reference [35]). The less intense rotational lines belong to F_5PY -H_2O, F_5PY -Ne and F_5PY -CH_2CO.

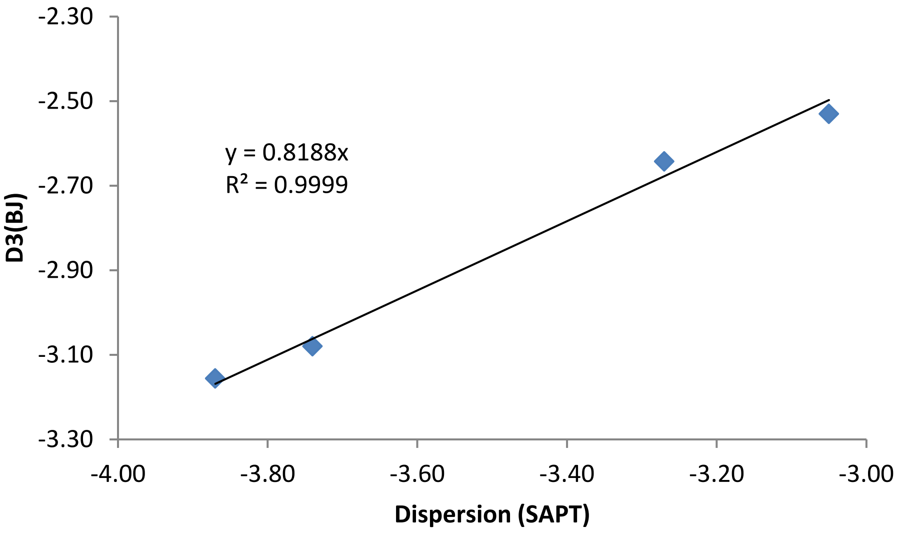

Figure S2. Correlation between the dispersion component of the DFT-SAPT energy and the D3(BJ) correction (kJ mol^{-1}).

Table S1. Rotational parameters predicted at B3LYP-D3BJ/6-311++G(2d,p) (DFT), MP2/6-311++G(2d,p) (MP2 1) MP2/aug-cc-pVTZ (MP2 2), and CCSD/6-311++G(2d,p) (CCSD) levels of theory for the perfluoropyridine···Ne complex.

Parameter[a]	DFT	MP2 1	MP2 2	CCSD
A/MHz	975.73	968.82	981.26	962.2
B/MHz	787.90	784.25	793.23	780.8
C/MHz	617.64	615.49	617.31	618.2
κ	-0.05	-0.04	-0.03	-0.05
P_{aa}/uÅ^2	470.86	471.93	470.38	469.7
P_{bb}/uÅ^2	347.39	349.17	348.30	347.7
P_{cc}/uÅ^2	170.56	172.48	166.73	177.5
3/2(χ_{aa})/MHz	3.297	2.974	2.982	3.11
1/4(χ_{bb}-χ_{cc})/MHz	-1.520	-1.403	-1.417	-1.51
μ_a/D	0.0	-0.1	-0.1	0.00
μ_b/D	1.0	1.1	1.1	-0.98
μ_c/D	0.2	0.0	0.0	-0.10
E/E$_h$	-873.6736835	-871.9124932	-872.2531675	-871.9035043

[a] A, B and C are the rotational constants. κ ray asymmetry parameter κ=(2B-A-C)/(A-C). $P_{\alpha\alpha}$ (α = a, b, c) are the planar moments of inertia, derived from, for example for P_{cc}=(I_a+I_b-I_c)/2. χ_{aa}, χ_{bb} and χ_{cc} are the ^{14}N quadrupole coupling constants. μ_a, μ_b and μ_c are the components of the electric dipole moment. E is the electronic energy in Hartree.

Table S2. Observed rotational parameters of pentafluoropyridine and those predicted at B3LYP-D3BJ/6-311++G(2d,p) (DFT), MP2/6-311++G(2d,p) (MP2 1) MP2/aug-cc-pVTZ (MP2 2), and CCSD/6-311++G(2d,p) (CCSD) levels of theory (data taken from reference [35].

Parameters[a]	parent	DFT	MP2 1	MP2 2	CCSD
A/MHz	1481.58184(19)[c]	1481.35	1477.87	1481.20	1484.36
B/MHz	1075.37335(17)	1073.22	1070.99	1074.22	1075.80
C/MHz	623.11194(16)	622.34	620.98	622.65	623.74
P_{aa} /uÅ^2	469.95288(16)	470.90	471.88	470.46	469.77
P_{bb} /uÅ^2	341.10379(16)	341.16	341.97	341.20	340.47
P_{cc} /uÅ^2	0.00399(16)	0.00	0.00	0.00	0.00
1.5(χ_{aa})/MHz	2.9496(31)	3.29	2.93	2.76	3.11
0.25(χ_{bb}-χ_{cc}) /MHz	-1.48514(91)	-1.58	-1.48	-1.46	-1.59
χ_{aa}/MHz	1.9664(53)	2.19	1.96	1.84	2.07
χ_{bb}/MHz	-3.9534(72)	-4.25	-3.94	-3.83	-4.22
χ_{cc}/MHz	1.9870(72)	2.05	1.98	1.99	2.14

[a] A, B and C are the rotational constants; χ_{aa}, χ_{bb} and χ_{cc} are the ^{14}N nuclear quadrupole coupling constants. $P_{\alpha\alpha}$ ($\alpha = a$, b or c) are the planar moments of inertia derived from the moments of inertia I_α as for example $P_c = (I_a+I_b-I_c)/2$.

Table S3. Principal inertial axis coordinates for the neon atom of pentafluoropyridine···Ne complex, analyzing the spectra with a Watson´s semirigid Hamiltonian with the A reduction and the I^r representation. The table compares the equilibrium coordinates (r_e) calculated at the B3LYP-D3BJ/6-311++G(2d,p) (DFT), MP2/6-311++G(2d,p) (MP2 1), MP2/aug-cc-pVTZ (MP2 2) and CCSD/6-311++G(2d,p) (CCSD) levels of theory with the experimental substitution (r_s) and effective (r_0) structures.

atom	method	*a*	*b*	*c*
**Ne**	r_s	[0.0000][a]	0.9672(15)[b]	2.75959(54)
	r_0	0.0000	0.910(24)	2.8134(64)
	$r_e^{\text{DFT 2}}$	0.0000	0.7366	2.7134
	$r_e^{\text{MP2 1}}$	0.0000	0.7940	2.7209
	$r_e^{\text{MP2 2}}$	0.0000	0.7789	2.6771
	r_e^{CCSD}	0.0000	0.814	2.757

[a] *a* coordinate is fixed to zero owing to the Kraitchman equations give imaginary values or cannot determinate the coordinates.

[b] Standard error is given in parentheses in units of the last digits.

Table S4. r_0 Geometry (Å, standard errors in parentheses) for pentafluoropyridine-Ne (see Figure 2).

N1,0.000(0.000),1.516(0.004),0.614(0.006)
C2,1.124(0.000),0.849(0.003),0.498(0.003)
C3,-1.124(0.000),0.849(0.003),0.498(0.003)
C4,1.196(0.000),-0.512(0.002),0.263(0.002)
c5,-1.196(0.000),-0.512(0.002),0.263(0.002)
c6,0.000(0.000),-1.203(0.002),0.143(0.005)
F1,2.258(0.000),1.537(0.004),0.617(0.006)
F2,-2.258(0.000),1.530(0.004),0.617(0.006)
F3,2.359(0.000),-1.151(0.002),0.152(0.005)
F4,-2.359(0.000),-1.151(0.002),0.152(0.005)
F5,0.000(0.000),-2.512(0.001),-0.083(0.011)
Ne,0.000(0.000),0.910(0.024),-2.813(0.006)

Table S5. Molecular graph, Optimized geometry (Å) and energy (hartree) at CCSD/6-311++G(2d,p) computational level for pentafluoropyridine-Ne

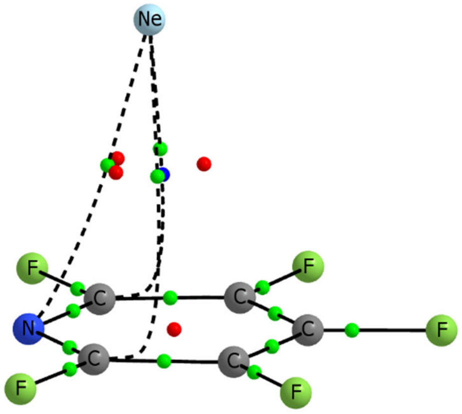

E(CCSD): -871.9035043

C,0.3337949593,-1.1578108603,0.
C,0.325679137,-0.4562539296,-1.1981969223
C,0.309564291,0.9286278233,-1.1260732809
N,0.3022663159,1.6020647193,0.
C,0.309564291,0.9286278233,1.1260732809
C,0.325679137,-0.4562539296,1.1981969223
F,0.2992293484,1.6237090292,2.2544931448
F,0.2992293484,1.6237090292,-2.2544931448
F,0.3298405854,-1.1006233752,-2.3604972089
F,0.3298405854,-1.1006233752,2.3604972089
F,0.3462296456,-2.4797404885,0.
Ne,-2.8700412474,0.3617551768,0.

Table S6. Molecular graph, Optimized geometry (Å) and energy (hartree) at CCSD/6-311++G(2d,p) computational level for pyridine-Ne

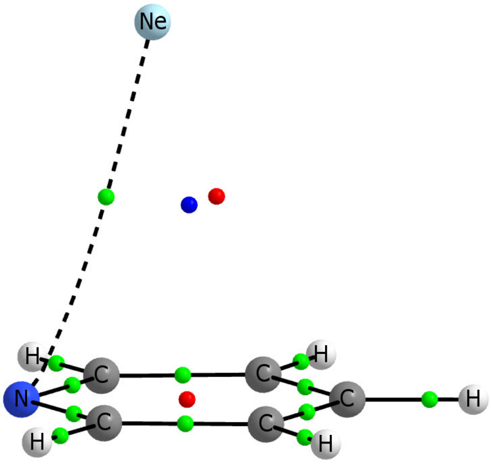

E(CCSD) = -376.4440756

C,0.3515709466,-1.2039813289,0.
C,0.3386480495,-0.4916695899,-1.1961413147
C,0.3126171302,0.9014894478,-1.1411064929
N,0.3001306356,1.6003277715,0.
C,0.3126171302,0.9014894478,1.1411064929
C,0.3386480495,-0.4916695899,1.1961413147
H,0.3032513498,1.4857462965,2.0577420931
H,0.3032513498,1.4857462965,-2.0577420931
H,0.3486443839,-0.9989831543,-2.1551129601
H,0.3486443839,-0.9989831543,2.1551129601
H,0.3728424254,-2.2894838225,0.
Ne,-2.9927958342,0.4167113797,0.

Table S7. Molecular graph, Optimized geometry (Å) and energy (hartree) at CCSD/6-311++G(2d,p) computational level for hexafluorobenzene-Ne

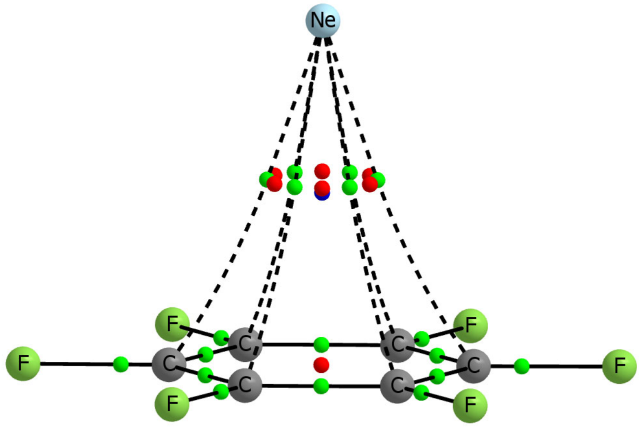

E(CCSD) = -954.9484181

C,0,1.388261,0.,0.
C,0,0.694131,-1.20227,0.
C,0,-0.694131,-1.20227,0.
C,0,-1.388261,0.,0.
C,0,-0.694131,1.20227,0.
C,0,0.694131,1.20227,0.
F,0,2.717914,0.,-0.003174
F,0,1.358957,-2.353783,-0.003174
F,0,-1.358957,-2.353783,-0.003174
F,0,-2.717914,0.,-0.003174
F,0,-1.358957,2.353783,-0.003174
F,0,1.358957,2.353783,-0.003174
Ne,0,0.,0.,-3.199147

Table S8. Molecular graph, Optimized geometry (Å) and energy (hartree) at CCSD/6-311++G(2d,p) computational level for benzene-Ne

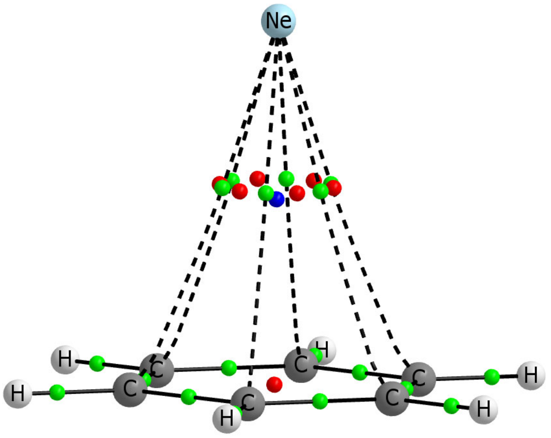

E(CCSD) = -128.7645537

C,0.00002,0.00029,1.39538
C,1.20842,0.00029,0.69770
C,1.20839,0.00029,-0.69766
C,-0.00002,0.00029-1.39537
C,-1.20843,0.00029-0.69771
C,-1.20836,0.000310.69764
H,0.00000,0.001942.48118
H,2.14875,0.001941.24058
H,2.14872,0.00195-1.24056
H,0.00000,0.00194-2.48117
H,-2.14877,0.00194,-1.24059
H,-2.14868,0.00186,1.24054
Ne,-0.00005,-3.31311,0.00003

Table S9. Observed rotational transitions and residuals (all the values in MHz) for the pentafluoropyridine···Ne^{20} complex in the ground vibrational state.

J′	K_{-1}′	K_{+1}′	J′′	K_{-1}′′	K_{+1}′′	F′	F′′	Obs.	Obs.-Cal.
**2**	0	2	1	1	1	1	0	2535.2347	-0.0102
						3	2	2536.9679	-0.0016
						1	1	2538.0573	0.0084
						2	1	2538.1593	-0.0057
**2**	1	2	1	0	1	2	1	2790.7516	-0.0001
						2	2	2791.3399	-0.0023
						1	1	2791.6369	-0.0003
						3	2	2791.9106	-0.0001
						1	0	2793.1140	0.0004
**2**	2	1	1	1	0	2	1	3436.9578	0.0009
						2	2	3437.4895	0.0022
						1	1	3437.9348	-0.0072
						3	2	3438.1204	0.0004
						1	0	3439.2638	-0.0044
**2**	2	0	1	1	1	1	0	3655.1998	0.0016
						2	2	3656.7623	-0.0013
						3	2	3656.8388	0.0008
						2	1	3657.8869	0.0020
						1	1	3658.0015	-0.0004
**3**	1	2	2	2	1	2	1	3821.9084	0.0006
						4	3	3822.6512	-0.0003
						3	2	3824.4014	-0.0010
**3**	0	3	2	1	2	3	3	3866.0556	-0.0028
						2	1	3866.2429	-0.0006
						4	3	3866.4295	-0.0007
						3	2	3866.6268	0.0000
						2	2	3867.1299	0.0008
**3**	1	3	2	0	2	3	2	3980.0504	0.0005
						3	3	3980.1286	0.0045
						4	3	3980.6955	0.0001
						2	2	3980.8228	0.0012
						2	1	3980.9383	0.0004
**3**	2	2	2	1	1	3	2	4671.1096	0.0012
						2	2	4671.1096	0.0012
						4	3	4672.3099	0.0003
						3	3	4672.3099	0.0003
						2	3	4672.3099	0.0003
						2	1	4672.9767	-0.0008
**4**	0	4	3	1	3	4	3	5140.9168	-0.0070
						3	2	5140.9168	-0.0070
						5	4	5140.9168	-0.0070
						3	3	5141.6871	0.0094

Table S9 (Continued).

J´	K_{-1}´	K_{+1}´	J´´	K_{-1}´´	K_{+1}´´	F´	F´´	Obs.	Obs.-Cal.
**4**	1	4	3	0	3	4	3	5179.4509	0.0004
						5	4	5179.7539	-0.0041
						3	2	5179.7539	-0.0041
						3	3	5180.2848	-0.0011
**4**	1	3	3	2	2	3	3	5319.3282	-0.0025
						5	4	5319.5164	-0.0010
						4	3	5320.2448	0.0019
						4	4	5320.2448	0.0019
**3**	3	1	2	2	0	3	3	5363.1553	-0.0035
						3	2	5363.2343	0.0011
						4	3	5363.8342	0.0007
						2	1	5364.0281	0.0007
						2	2	5364.1468	0.0024
**3**	2	1	2	1	2	2	1	5405.8997	-0.0018
						4	3	5406.5788	0.0008
						3	2	5408.1733	-0.0013
**3**	3	0	2	2	1	3	3	5449.7775	-0.0040
						2	1	5450.1434	0.0002
						4	3	5450.3110	0.0011
						3	2	5450.4143	0.0000
						2	2	5451.1252	-0.0030
**4**	2	3	3	1	2	4	3	5830.4816	-0.0003
						5	4	5831.4839	0.0001
						3	2	5831.8457	0.0000
**5**	0	5	4	1	4	5	4	6388.4309	-0.0024
						4	3	6388.5230	0.0156
						6	5	6388.5230	0.0156
**5**	1	5	4	0	4	5	4	6399.4479	-0.0005
						4	3	6399.5909	0.0002
						6	5	6399.5909	0.0002
**5**	2	3	4	3	2	4	3	6522.5187	-0.0114
						6	5	6522.7830	0.0011
						5	4	6524.0279	-0.0042
**4**	3	2	3	2	1	4	3	6631.9955	-0.0007
						5	4	6632.9637	0.0005
						4	4	6633.0204	-0.0041
						3	2	6633.3059	-0.0013
**5**	1	4	4	2	3	6	5	6719.9613	0.0026
						5	4	6720.1395	0.0009
**5**	2	4	4	1	3	5	4	6962.4784	-0.0008
						6	5	6963.1574	0.0007
						4	3	6963.3320	-0.0017

Table S9 (Continued).

J′	K-1′	K+1′	J′′	K-1′′	K+1′′	F′	F′′	Obs.	Obs.-Cal.
**4**	3	1	3	2	2	3	3	7032.4541	-0.0020
						5	4	7032.5968	0.0000
						4	3	7033.1412	-0.0045
						4	4	7033.1412	-0.0045
**4**	4	1	3	3	0	4	3	7273.2836	0.0016
						5	4	7273.5410	-0.0064
						3	2	7273.5410	-0.0064
**4**	4	0	3	3	1	4	3	7298.6876	0.0036
						3	2	7298.7587	0.0088
						5	4	7298.7587	0.0088
**4**	2	2	3	1	3	5	4	7375.4872	0.0064
**6**	0	6	5	1	5	6	5	7626.3135	0.0005
						5	4	7626.3722	0.0001
						7	6	7626.3862	0.0000
**6**	1	6	5	0	5	6	5	7629.1647	0.0010
						5	4	7629.2390	-0.0006
						7	6	7629.2521	0.0011
						5	5	7630.1374	-0.0003
**5**	3	3	4	2	2	5	4	7795.1912	-0.0041
						6	5	7796.2135	0.0002
						4	3	7796.4885	-0.0054
**6**	6	0	6	5	1	6	6	2537.5150	-0.0027
**6**	1	5	5	2	4	6	5	8031.5368	0.0036
						7	6	8031.5781	-0.0031
						5	4	8031.5900	-0.0010
**6**	2	4	5	3	3	5	4	8109.2641	0.0000
						7	6	8109.3619	0.0003
						6	5	8110.0098	-0.0001
**6**	2	5	5	1	4	6	5	8123.3773	0.0011
						7	6	8123.7524	0.0008
						5	4	8123.8254	0.0000
**5**	4	2	4	3	1	5	4	8608.9656	0.0000
						6	5	8609.5625	0.0000
						4	3	8609.7130	-0.0002
**5**	4	1	4	3	2	4	3	8774.0145	-0.0011
						6	5	8774.0236	0.0000
						5	4	8774.0782	-0.0002
**5**	3	2	4	2	3	4	3	8826.6898	0.0015
						6	5	8826.9026	-0.0001
						5	4	8827.9852	-0.0001
**7**	0	7	6	1	6	7	6	8861.2692	0.0002
						6	5	8861.3167	0.0009
						8	7	8861.3258	-0.0020

Table S9 (Continued).

J′	K_{-1}′	K_{+1}′	J′′	K_{-1}′′	K_{+1}′′	F′	F′′	Obs.	Obs.-Cal.
**7**	1	7	6	0	6	7	6	8861.9628	0.0004
						6	5	8862.0123	-0.0003
						8	7	8862.0229	-0.0012
**6**	3	4	5	2	3	6	5	8884.4278	0.0003
						7	6	8885.3263	-0.0003
						5	4	8885.5242	-0.0003
**5**	5	1	4	4	0	5	4	9163.5408	-0.0001
						4	3	9163.6603	0.0003
						6	5	9163.6736	0.0002
**5**	5	0	4	4	1	5	4	9169.7533	0.0006
						4	3	9169.8242	-0.0007
						6	5	9169.8483	0.0005

Table S10. Observed rotational transitions and residuals (all the values in MHz) for the pentafluoropyridine···Ne^{22} complex in the ground vibrational state.

J′	K_{-1}′	K_{+1}′	J′′	K_{-1}′′	K_{+1}′′	F′	F′′	Obs.	Obs.-Cal.
**2**	0	2	1	1	1	1	0	2518.0203	0.0029
						3	2	2519.7182	0.0000
						2	1	2520.9202	0.0038
**2**	1	2	1	0	1	2	1	2757.6061	0.0020
						2	2	2758.1936	-0.0022
						3	2	2758.7332	-0.0014
						1	0	2759.9207	-0.0018
**2**	2	1	1	1	0	2	1	3349.4072	-0.0018
**2**	2	0	1	1	1	2	2	3545.6112	-0.0021
						3	2	3545.7162	-0.0005
						2	1	3546.7102	0.0020
						1	1	3546.8731	0.0027
**3**	1	2	2	2	1	4	3	3786.1120	-0.0045
						3	2	3787.8632	0.0054
**3**	0	3	2	1	2	2	1	3839.0336	0.0032
						4	3	3839.2154	-0.0002
						3	2	3839.4263	0.0005
						2	2	3839.8658	-0.0038
**3**	1	3	2	0	2	3	2	3947.8273	0.0000
						3	3	3947.9329	0.0021
						4	3	3948.4646	0.0007
						2	2	3948.5512	0.0035
						2	1	3948.7095	0.0003
**3**	2	2	2	1	1	2	2	4580.5738	-0.0008
						4	3	4581.7481	0.0010
						3	3	4581.7481	0.0010
						2	3	4581.7481	0.0010
						2	1	4582.4015	0.0022
**4**	0	4	3	1	3	3	2	5108.6517	-0.0042
						4	3	5108.6517	-0.0042
						5	4	5108.6517	-0.0042
						3	3	5109.3480	-0.0081
**4**	1	4	3	0	3	4	4	5146.0525	-0.0029
						4	3	5146.3845	0.0004
						5	4	5146.6841	-0.0073
						3	2	5146.6841	-0.0073
						3	3	5147.1612	-0.0020
**3**	3	1	2	2	0	3	3	5214.2624	0.0030
						3	2	5214.3630	0.0002
						4	3	5214.9434	0.0008
						2	1	5215.1232	-0.0001
						2	2	5215.2822	-0.0033

Table S10 (Continued).

J´	K_{-1}´	K_{+1}´	J´´	K_{-1}´´	K_{+1}´´	F´	F´´	Obs.	Obs.-Cal.
**3**	2	1	2	1	2	2	1	5236.7585	-0.0017
						4	3	5237.4084	0.0029
						3	2	5238.9279	-0.0026
**4**	1	3	3	2	2	3	3	5258.0799	-0.0002
						3	4	5258.0799	-0.0002
						5	4	5258.2714	0.0008
						4	3	5259.0102	-0.0003
						4	4	5259.0102	-0.0003
**3**	3	0	2	2	1	3	3	5289.9141	0.0085
						2	1	5290.2908	-0.0022
						4	3	5290.4533	-0.0003
						3	2	5290.5403	0.0006
						2	2	5291.2867	0.0065
**4**	2	3	3	1	2	3	2	5745.5654	0.0015
**5**	0	5	4	1	4	5	4	6352.7972	-0.0039
						6	5	6352.8841	0.0067
**5**	1	5	4	0	4	5	4	6363.8558	-0.0017
						4	3	6363.9984	-0.0006
						6	5	6363.9984	-0.0006
**4**	3	2	3	2	1	4	3	6477.9885	-0.0004
						3	2	6479.2595	-0.0005
**5**	1	4	4	2	3	4	3	6645.5476	0.0015
						6	5	6645.5476	0.0015
						5	4	6645.7533	-0.0064
**4**	3	1	3	2	2	3	3	6829.2932	-0.0030
						5	4	6829.4200	-0.0054
						4	3	6829.9306	0.0009
**5**	2	4	4	1	3	5	4	6881.4205	0.0013
						6	5	6882.0915	-0.0007
						4	3	6882.2687	-0.0004
**4**	4	1	3	3	0	4	3	7063.6664	-0.0017
						5	4	7063.9193	-0.0029
						3	2	7063.9193	-0.0029
**4**	4	0	3	3	1	5	4	7085.3709	-0.0074
**6**	0	6	5	1	5	6	5	7587.6707	0.0004
						5	4	7587.7271	-0.0002
						7	6	7587.7409	0.0006
**6**	1	6	5	0	5	6	5	7590.6043	0.0012
						5	4	7590.6780	-0.0004
						7	6	7590.6893	0.0009
**5**	3	3	4	2	2	5	4	7646.3956	0.0013
						6	5	7647.3867	-0.0001
						4	3	7647.6637	0.0031

Table S10 (Continued).

J´	K_{-1}´	K_{+1}´	J´´	K_{-1}´´	K_{+1}´´	F´	F´´	Obs.	Obs.-Cal.
**6**	1	5	5	2	4	6	5	7951.4185	-0.0004
						7	6	7951.4537	0.0004
						5	4	7951.4617	0.0003
**6**	2	4	5	3	3	5	4	8000.0828	0.0000
						7	6	8000.1841	0.0002
						6	5	8000.8514	-0.0003
**6**	2	5	5	1	4	6	5	8043.0804	0.0003
						7	6	8043.4573	0.0001
						5	4	8043.5320	-0.0002
**5**	4	2	4	3	1	5	4	8388.1727	0.0013
						6	5	8388.7419	-0.0005
						4	3	8388.8843	-0.0012
**5**	4	1	4	3	2	4	3	8529.4084	-0.0013
						6	5	8529.4155	0.0014
						5	4	8529.4484	0.0004
**5**	3	2	4	2	3	4	3	8556.1963	-0.0004
						6	5	8556.3985	-0.0001
						5	4	8557.4230	0.0008
**7**	0	7	6	1	6	7	6	8819.6410	0.0000
						6	5	8819.6865	-0.0001
						8	7	8819.6986	0.0009
**7**	1	7	6	0	6	7	6	8820.3717	-0.0003
						6	5	8820.4204	-0.0011
						8	7	8820.4314	-0.0006
**6**	3	4	5	2	3	6	5	8746.6323	-0.0006
						7	6	8747.5184	0.0006
						5	4	8747.7120	-0.0004
**5**	5	1	4	4	0	5	4	8895.1297	0.0002
						4	3	8895.2428	-0.0006
						6	5	8895.2593	0.0009
**5**	5	0	4	4	1	5	4	8900.2795	0.0000
						4	3	8900.3520	0.0004
						6	5	8900.3742	-0.0006
